# Quenching Sensitivity Study of New High-Strength Aluminum Alloys Based on an Immersion End-Quenching and Step-Quenching Technique

**DOI:** 10.3390/ma18133132

**Published:** 2025-07-02

**Authors:** Chengbo Li, Qinyao Chen, Yiming Qin, Puli Cao, Shusheng Lin, Donghua Lan, Wenhui Huang, Wang Zhou, Wengang Chen

**Affiliations:** 1 School of Mechanical Engineering and Mechanics, Xiangtan University, Xiangtan 411105, China; csulicb@163.com (C.L.); 18627457239@163.com (Q.C.); 243101042@csu.edu.cn (P.C.); 2 College of Traffic and Transportation, Nanning University, Nanning 530200, China; 3 School of Materials Science and Engineering, Central South University, Changsha 410083, China; 4 Liuzhou Chenglong Special Purpose Vehicle Co., Ltd., Liuzhou 545006, China; zcing0106@163.com; 5 Guangxi Guangtou Liuzhou Aluminum Co., Ltd., Liuzhou 545001, China; 15873214198@163.com (D.L.); 15573996454@163.com (W.H.); 6 Guangdong Hoshion Industrial Aluminium Co., Ltd., Zhongshan 528463, China; 18670956332@163.com; 7 Dongfeng Liuzhou Motor Co., Ltd., Liuzhou 545006, China; 18670956331@163.com

**Keywords:** high-strength aluminum alloy, quenching sensitivity, immersion end-quenching, step-quenching, microstructure

## Abstract

Based on end-quenching and step-quenching experiments combined with scanning electron microscopy (SEM) and transmission electron microscopy (TEM), the quench sensitivity of a novel high-strength aluminum alloy was investigated and compared with that of GB/T 7075 and 7175 alloys; quench factor analysis (QFA) was employed to predict the hardness values of the alloy and investigate the effect of quenching rate on its mechanical properties. The experimental results indicate that when the cooling rate decreases from 402.5 °C/s to 3.6 °C/s, the hardness reduction rate of the novel high-strength aluminum alloy is 15%. Furthermore, the nose temperature of the time–temperature–property (TTP) curve for this alloy is 325 °C, with a critical transformation time of 0.4 s. The quench-sensitive temperature range is 219 °C to 427 °C, which is lower than the quenching sensitivity of 7075 and 7175 alloys. The new alloy reduces its quenching sensitivity by optimizing the composition of alloying elements. Furthermore, the QFA demonstrates high predictive accuracy, with a maximum error of 5%. The smaller the quenching factor τ, the greater the hardness of the alloy after aging. Combined with the TTP curve, the alloy properties are optimized by modulating the quenching rate. This study provides a theoretical basis for selecting hot forming–quenching integrated process parameters in automotive high-strength aluminum alloys.

## 1. Introduction

The 7XXX series aluminum alloys, characterized by their low density, high strength, and excellent processing properties, have become integral components in the automotive industry, aerospace engineering, and advanced weaponry fabrication [1,2,3,4]. With the progressive expansion of aerospace and related technological domains, aircraft manufacturing has been increasingly moving toward “large-scale” development. To achieve structural weight reduction, lower manufacturing costs, and extend service life, the requirements of the specific strength, fracture toughness, and corrosion resistance of aluminum alloys have been continuously increasing [5,6]. With the widespread application of high-performance large structural components, the demand for thick-section 7XXX series aluminum alloy plates with superior comprehensive properties has been growing. The 7XXX series aluminum alloys are well known to have high quenching sensitivity. When the alloy material undergoes quenching after solution treatment, a decrease in the quenching rate leads to the precipitation of numerous coarse equilibrium phases within the grains. These phases exhibit negligible strengthening effects but extensively consume Zn and Mg elements in the matrix. They reduce the supersaturation of the matrix and then significantly inhibit the precipitation of strengthened phases during the subsequent aging, which ultimately leads to a decrease in the strength, hardness, and corrosion resistance of the aging material [7,8,9]. When conducting solution treatment for the thick plates of these alloys, the cooling rate is unevenly distributed along the thickness direction, resulting in different cooling rates between the core and the surface, which causes inhomogeneity in microstructure and properties across the thickness [10]. This is a critical technical challenge in the fabrication of thick-section materials. In the quenching process of 7XXX series aluminum alloy thin plates, excessively high cooling rates can cause warping deformation, while excessively low cooling rates can lead to significant performance losses. Currently, some high-end automotive pillars employ the GB/T 7075 [11] aluminum alloy hot forming–quenching integrated technology, but issues such as insufficient cooling intensity during the forming process can result in performance degradation. Therefore, it is essential to research 7XXX series aluminum alloys regarding their quenching sensitivity.

Nowadays, many studies have been reported on the quench sensitivity of 7XXX series aluminum alloys. Zhou et al. [1] investigated 7xxx series aluminum alloys for aircraft applications. They found these alloys exhibit high specific strength, high toughness, and outstanding workability but suffer from high stress corrosion susceptibility and stringent quenching requirements. Optimizing alloy composition and improving heat treatment processes are key approaches to address these issues. Li et al. [12] determined the continuous cooling transformation (CCT) curve of 7475 [13] aluminum alloys through resistivity measurements, X-ray diffraction analysis, and quantitative microstructural analysis, concluding that the critical cooling rate for the experimental alloy is 100–110 °C/s. Sujoy et al. [14] investigated the effect of elements on the quenching sensitivity of Al-Si-Mg series aluminum alloys. It was found that the quenching factor increases with decreasing quenching rate, and the higher the quenching sensitivity, the larger the quenching factor overall. Azam et al. [15] found that 7xxx series aluminum alloys exhibit high stacking fault energy, making it difficult to obtain ultrafine-grained structures through conventional processing. However, adding Sc and Zr elements can refine their microstructure and enhance overall performance. Lin et al. [16] constructed a time–temperature–property (TTP) curve of 7475 aluminum alloys based on step-quenching experiments. Wang et al. [17] investigated the quench sensitivity and isothermal precipitation behavior of 7075 aluminum alloy and obtained its TTP curve. Ma et al. [18] established the TTP curve for AA7136 [11] aluminum alloy, identifying a nose temperature of 346 °C and a corresponding transformation time of 0.245 s. Liu et al. [19] compared the TTP curves of typical aluminum alloys and found that AA7075, AA7175 [11], and AA7055 [11] alloys exhibit high quench sensitivity, while AA7085 [11] alloy demonstrates the lowest quench sensitivity. Dolan et al. [20] conducted in-depth research on the quench sensitivity of 7175, 6061 [21], and 2017A [21] aluminum alloys and plotted their respective TTP curves. Although there are numerous current studies on the quench sensitivity of 7XXX aluminum alloys, there are fewer studies on predicting the properties of 7XXX series aluminum alloys, solving the quench sensitivity problem, and improving the properties of the alloys. To address the quench sensitivity issues in the hot forming process of automotive 7075 aluminum alloy, in this study, the authors developed a novel high-strength 7XXX series aluminum alloy. Using this alloy as the research subject, continuous variations in cooling rates were achieved through an intrusive end-quenching experimental method, enabling the investigation of properties and microstructures under different cooling rates. Additionally, the hardness of the alloy at different temperatures was also obtained by step-quenching experiments, thereby constructing a TTP curve and identifying the quench-sensitive temperature range. This lays the theoretical foundation for research on the hot forming–quenching integrated process technology of this novel alloy.

## 2. Experimental Materials and Methods

### 2.1. Experimental Material

This study employs a 3 mm thick novel high-strength aluminum alloy sheet. Its chemical composition (Table 1) is analyzed using a SPECTROMAXx (LMX07) spectrometer from SPECTRO, Kleve, Germany.

### 2.2. Immersion End-Quenching Experiment

**Figure 1:**
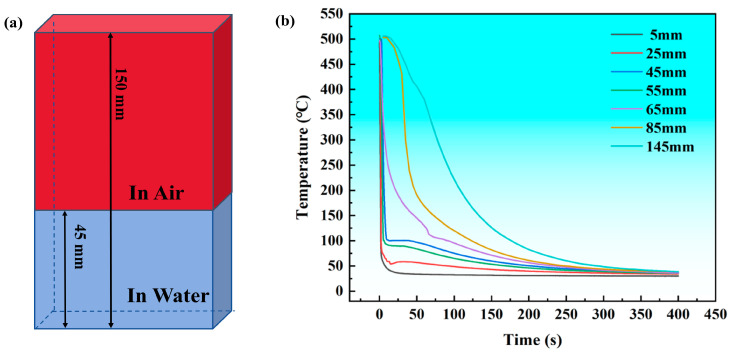
Schematic diagram and measured cooling curve for immersion end-quenching. (**a**) schematic diagram; (**b**) measured cooling curve.

The samples are in 150 mm in length and have a cross-section size of 80 × 3 mm. Firstly, they are put into a SX2-4-10 type box-type resistance furnace (from Shanghai, China) for solid solution heat treatment; the solid solution temperature is 475 °C, and the holding time is 2 h. Subsequently, an end of the sample is quickly immersed in water at about 25 °C. The depth of the sample’s immersion in the water is 45 mm, leaving 105 mm exposed to air (Figure 1a), and the cooling process is approximately 400 s. Small holes of 1.5 mm in diameter and 10mm in depth along the *x*-axis are drilled at different locations in the cross-section of the plate. Thermocouples are embedded in these holes, and temperature–time curves are recorded using a QT-6-K high-precision pyrometer (from Guangdong, China) during quenching. The measured cooling curves are obtained as shown in Figure 1b. Based on the measurements, the cooling rates of the alloy at end distances of 5 mm, 55 mm, 85 mm, and 145 mm are calculated to be 402.5 °C/s, 96.4 °C/s, 10.6 °C/s, and 3.6 °C/s, respectively. After the samples are cooled to room temperature, artificial aging is carried out for 24 h at 120 °C in an electrically heated blast-drying oven type 101-2BS (from Shanghai, China).

### 2.3. Step-Quenching Experiment

**Figure 2:**
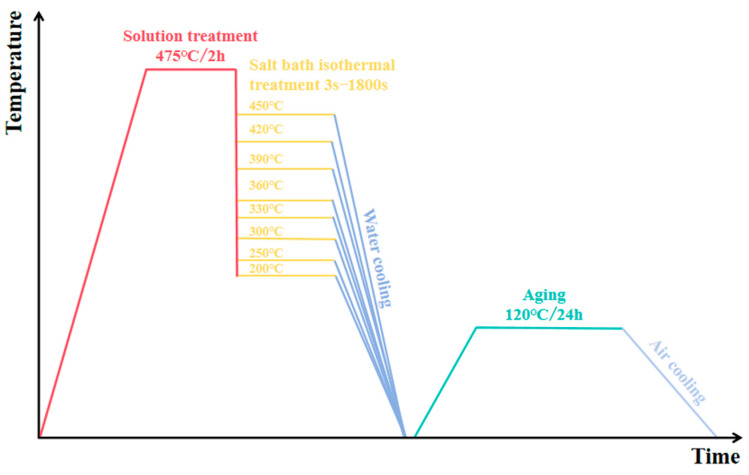
Step-quenching experimental process.

Figure 2 illustrates the experimental process chart of step-quenching. Square samples are placed in an SX2-4-10 box-type resistance furnace at 475 °C for 2 h of solid solution heat treatment. After that, salt bath isothermal quenching is carried out immediately in the SG-5-12 resistance furnace (from Shanghai, China), with holding temperatures ranging from 200 °C to 450 °C and durations of 3–1800 s. Upon completion of isothermal holding, the samples are immediately immersed in water at room temperature for quenching. Once the samples are cooled to 25 °C, they are subjected to artificial aging in the 101-2BS electric blast-drying oven at 120 °C for 24 h. Step-quenching experiments establish the TTP profile for the alloy. This curve is then compared with those of other alloys to assess the level of quenching sensitivity.

### 2.4. Hardness Measurement

Three sets of parallel samples are prepared in each experiment to ensure data accuracy. At least five hardness measurements are recorded at each test position, with the final results averaged. According to GB/T 4340.3-2012 [22], the load is set to 10 N, and the holding time is maintained at 15 s. In the immersion end-quenching experiment, the first hardness measurement point is located 5 mm from the quenched end, followed by a hardness value at 10 mm intervals.

### 2.5. Microstructure Analysis

Samples are collected at various sites for microstructure investigation. First, we sequentially grind the sample surface using water abrasive sandpaper to remove the oxide layer and then use metallographic sandpaper on it until 1200# to brighten the surface, before then mechanically polishing it. A TESCAN MIRA4 LMH scanning electron microscope (from Brno, Czech Republic) is used to examine the microstructure, and the voltage is 20 keV. Transmission electron microscopy (TEM, Titan G2 60-300, from Hillsboro, OR, USA) is used to observe the quenching and aging precipitation phases. The samples are polished to a thickness of approximately 80 μm, stamped into discs of a 3 mm diameter, and then thinned by double-jet electropolishing in a solution of 80% methanol and 20% nitric acid. The temperature of the electrolyte is maintained at approximately −25 °C using liquid nitrogen.

## 3. Results

### 3.1. Immersion End-Quenching Experiment

**Figure 3:**
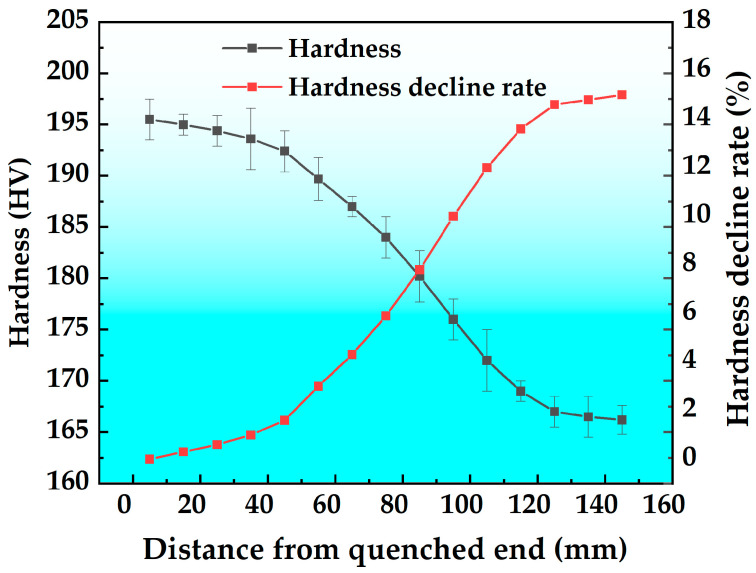
Hardness curve and hardness reduction rate curves.

The hardness curve and hardness reduction rate curve of the T6 state alloy are plotted in Figure 3. According to the figure, the hardness of the alloy decreases with increasing distance from the quenched end. Based on the hardness curve, using the hardness value at a distance of 5 mm from the end as a reference, the hardness reduction rate was calculated, yielding the hardness reduction rate curve. As the results illustrate, the hardness reduction rate increases with increasing quenched end distance. Within the ranges of 5–45 mm and 115–145 mm from the quenched end, the hardness reduction is minimal, with reduction rates of 1.6% and 1.4%, respectively. However, within the range of 45–115 mm from the quenched end, the hardness reduction is more significant, with a reduction rate of 12%. At a distance of 145 mm from the quenched end, the hardness reduction rate reaches its maximum value of 15%. This shows that the alloy has a high quenching sensitivity.

**Figure 4:**
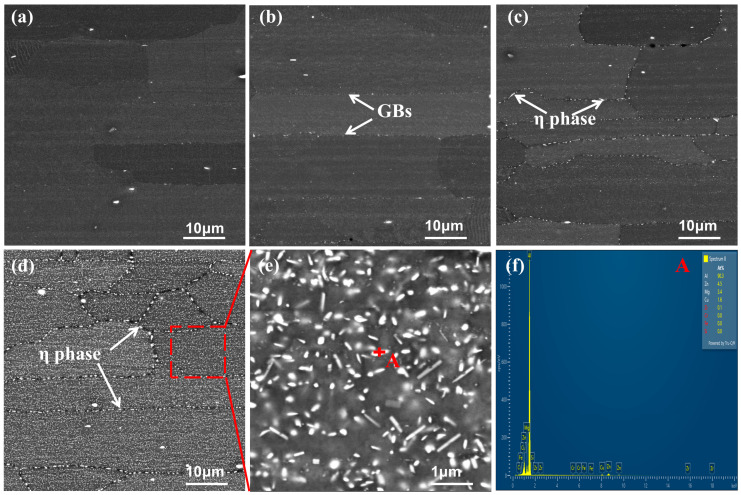
SEM images with varying cooling rates. (**a**) 402.5 °C/s (**b**) 96.4 °C/s (**c**) 10.6 °C/s (**d**,**e**) 3.6 °C/s (**f**) EDS.

Figure 4 presents the SEM micrographs of the T6 state alloy at different cooling rates. As depicted in the figure, as the rate of quenching decreases, bright precipitates preferentially form at grain boundaries and subsequently gradually precipitate within the grains. At the highest cooling rate of 402.5 °C/s (Figure 4a), there are some coarse second phases in the grain, and the EDS result is (wt.%): Al-16.5Fe-4.4Cu, which is known in the literature [23] as the iron-rich phase (Al_7_Cu_2_Fe) formed during the melting and casting. However, at this time, no obvious quenching precipitation phases are observed either at grain boundaries or within the grains. At a cooling rate of 96.4 °C/s, bright precipitates become visible at the grain boundaries (Figure 4b). When the cooling rate is further reduced to 10.6 °C/s (Figure 4c), the bright precipitates at the grain boundaries become more pronounced, and smaller quenching precipitates begin to form within the grains. At the slowest cooling rate of 3.6 °C/s, coarse precipitates are densely distributed at the grain boundaries and within the grains (Figure 4d,e). The EDS result of Figure 4f is (wt.%):Al-4.5Zn-3.4Mg-1.6Cu, which shows that the rod-shaped bright phase is the quenching precipitation phase η phase (MgZn_2_).

**Figure 5:**
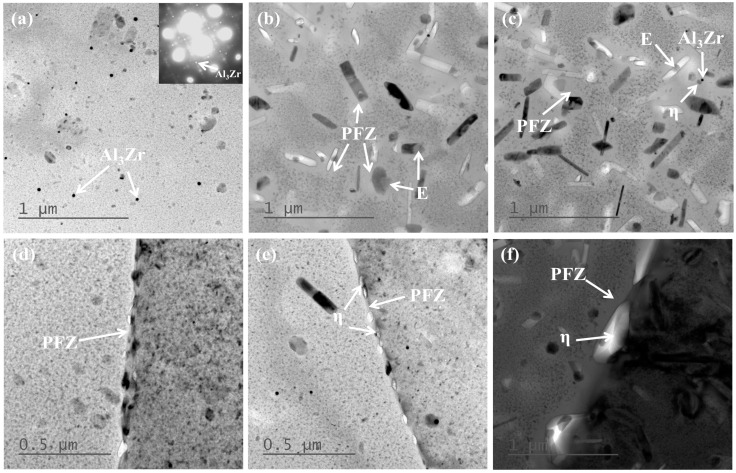
TEM images with varying cooling rates. (**a**,**d**) 402.5 °C/s (**b**,**e**) 10.6 °C/s (**c**,**f**) 3.6 °C/s.

Figure 5 illustrates the TEM micrographs of the T6 state alloy at various cooling rates. It is evident that the size and quantity of quenching precipitates increase as the cooling rate decreases. When the cooling rate is 402.5 °C/s (Figure 5a,d), irregularly shaped particles are observed within the grains and have an EDS (wt.%) result of Al-12.3Mg-4.6Cr, being identified as the E phases (Al_18_Cr_2_Mg_3_). Simultaneously, spherical particles are present in the grains, which are determined to be Al_3_Zr particles based on the diffraction pattern in Figure 5a. At this time, fine, uniformly dispersed aging strengthening phases appear within the grains. Discontinuously distributed aging precipitates are observed at the grain boundaries, and the precipitate-free zone (PFZ) is relatively narrow. As the cooling rate decreases to 10.6 °C/s, quenching precipitates are observed both within the grains (associated with the E phase) and at the grain boundaries. At this stage, distinct PFZs are observed around the quenching precipitates and grain boundaries, as illustrated in Figure 5b,e. When the cooling rate is further reduced to 3.6 °C/s, a large number of coarse quenching precipitates form both within the grains and at the grain boundaries, and the PFZ width significantly increases, which is displayed in Figure 5c,f. Rod-shaped quenching precipitates are observed on both the E phase and Al_3_Zr particles. Additionally, the aging precipitation phases become larger in size but fewer in number, resulting in a weakened strengthening effect and reduced hardness.

### 3.2. Step-Quenching Experiment

**Figure 6:**
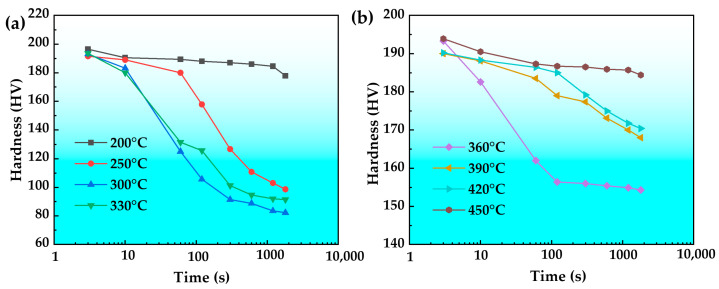
Hardness of the T6 state high-strength aluminum alloy with isothermal holding curves. (**a**) 200–330 °C (**b**) 360–450 °C.

Figure 6 illustrates the relationship between the hardness of the T6 state alloy and isothermal holding time. From the figure, with the increase in the isothermal holding time, the hardness of the alloy decreases. The hardness generally decreases with increasing insulation temperature in the range of 200–330 °C. When the holding temperatures are 300 °C and 330 °C, the hardness decreases by 56.9% and 52.8%, respectively. However, at a holding temperature of 200 °C, the hardness remains relatively stable despite the holding time variation, and the overall reduction rate is just 9.4%. In the holding temperature range of 360–450 °C, the hardness generally increases with increasing holding temperature but decreases with extended holding time. Overall, the hardness shows a trend of first decreasing rapidly and then slowly, but the reduction rate is different. At 360 °C holding, the hardness decreases fast until 60 s, after which the reduction rate slows down, and the overall reduction rate is 20.2%. When holding at 450 °C, the hardness shows a relatively regular slow decrease with the extension of holding time, with a decrease rate of only 4.8%. It is evident that for the alloy during the isothermal treatment process, the hardness in the middle temperature zone of 250–390 °C declines rapidly, and the most rapid decline in hardness occurs within the range of 300–330 °C.

**Figure 7:**
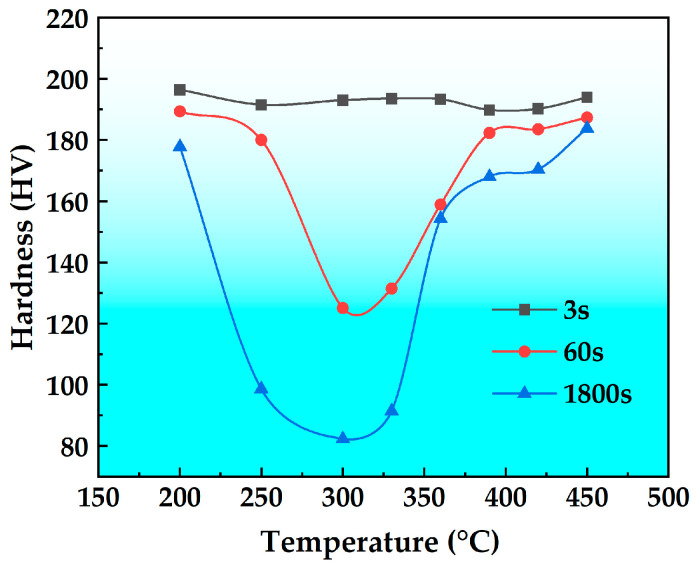
Isochronal hardness curve of the T6 state high-strength aluminum alloy.

Figure 7 shows the isochronal hardness curve of the T6 state alloy. As shown in the figure, the hardness exhibits minimal variation at a holding time of 3 s. However, at holding times of 60 s and 1800 s, the hardness first decreases and then increases with increasing holding temperature, forming a characteristic U-shaped curve. The hardness reaches its minimum value of 125 HV at 60 s and 82 HV at 1800 s, both at a holding temperature of 300 °C.

Based on the measured TTP curve, the fitting is performed using the following formula.

(1) $$C(T)=-k_{1}k_{2}exp\frac{k_{3}k_{4}^{2}}{RT{\left(k_{4}-T\right)}^{2}}exp(\frac{k_{5}}{RT}),$$

**Figure 8:**
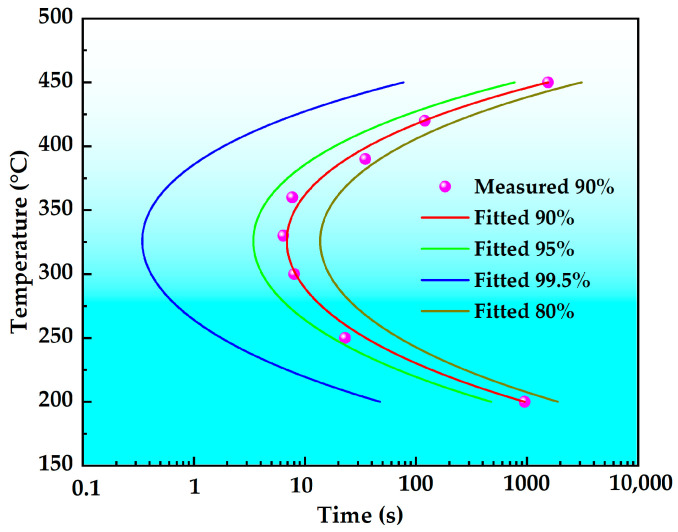
TTP diagrams of the high-strength aluminum alloy.

In Equation (1), C(T) represents the critical time (s) needed to precipitate a specific amount of solute. The constant k_1_ is defined as (1 − ζ), where ζ represents the natural logarithm of the untransformed fraction during quenching. K_2_ is a constant related to the reciprocal of the nucleation number, and k_3_ is a constant associated with the energy required for nucleation (J·mol^−1^). K_4_ is a constant related to the dissolution temperature (K), and k_5_ is a constant associated with the activation energy for diffusion (J·mol^−1^). R is the gas constant (J·mol^−1^·K^−1^), and T is the temperature (K). According to the data in Figure 6, the TTP curves for the peak hardness to decrease to 99.5%, 95.0%, 90.0% and 80% in the T6 state alloy can be obtained by applying Equation (1), as shown in Figure 8. It is generally accepted that the time required for the peak hardness to drop to 99.5% is the critical transition time [24]. There is a general belief that the shorter the critical transition time, the worse the stability of the solid solution. If taking the TTP curve when the peak hardness decreases to 99.5% as a standard, the nose temperature is about 325 °C, the critical transition time is about 0.4 s, and the corresponding quenching sensitivity temperature range for an isothermal time of 10 s is 219–427 °C. In general, for mechanical properties, a larger quench sensitivity temperature range, a higher nose temperature, and a shorter transformation time indicate higher quenching sensitivity of the material.

**Figure 9:**
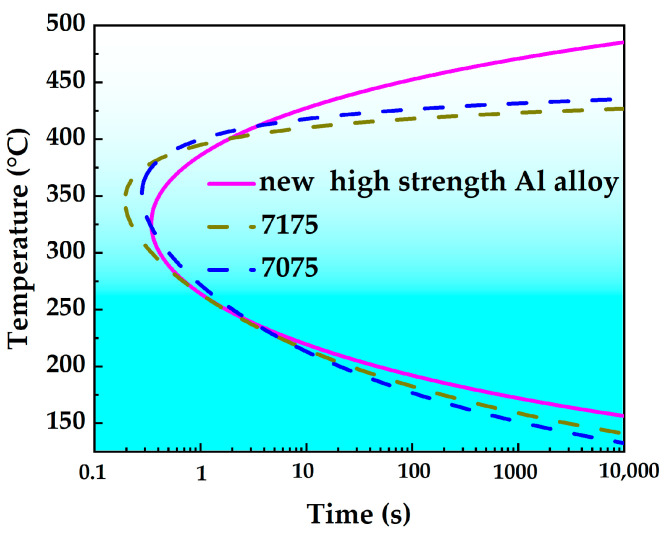
TTP curves of three 7XXX series aluminum alloys.

Taking the TTP curve with peak hardness reduced to 99.5% as a standard, the TTP curves of 7175, 7075 and the new high-strength aluminum alloys are plotted together for comparison, as shown in Figure 9. As shown in the figure, the new high-strength aluminum alloy curve is on the rightmost side, followed by 7075 and 7175 aluminum alloys from right to left. The fitted corresponding constants are shown in Table 2, and their corresponding nose temperatures, quench sensitivity temperature range, and transformation times are shown in Table 3. Among them, the nose temperatures of 7175, 7075 and the new high-strength aluminum alloy are 340 °C, 350 °C, and 325 °C, with corresponding critical transition times of 0.16 s, 0.23 s and 0.4 s, respectively. With a holding time of 10 s to define the quench sensitivity temperature range, the quench sensitivity temperature ranges for 7175, 7075, and the new high-strength aluminum alloy are 197 °C, 215 °C, and 208 °C, respectively. In summary, the new high-strength aluminum alloy demonstrates the lowest quenching sensitivity among the three alloys.

## 4. Analysis and Discussion

The 7XXX series aluminum alloys are heat-treatable strengthening alloys [1]. Through rapid cooling after solution treatment and subsequent aging treatment, fine and uniformly distributed second-phase particles can be formed in the matrix. These high-density precipitation strengthening phases significantly increase the strength and hardness of the alloy. After solid solution treatment, the matrix has a relatively high concentration of solute atoms. During slow quenching, quenching precipitated phases will gradually precipitate as the temperature decreases [25,26,27,28], and these precipitated phases are easier to precipitate at some defects of the alloy (grain boundaries, phase boundaries, etc.), as shown in Figure 4 and Figure 5. Equilibrium phases preferentially precipitate at grain boundaries due to the higher energy at these sites, which reduces the nucleation activation energy of the second phase. Additionally, the precipitation of the second phase is closely related to the cooling rate, as depicted in Figure 4 and Figure 5. The smaller the cooling rate, the more quenching precipitated phases during cooling, and the larger the size. These quenching precipitation phases not only lack a strengthening effect but also consume significant amounts of Mg and Zn elements, substantially reducing the concentration of these elements in the aluminum matrix. This leads to a decrease in the supersaturation and vacancy concentration of solute atoms in the matrix. During the aging process, the critical nucleation activation energy and critical nucleation radius of the aging precipitated phases increase, making it difficult for the aging precipitated phases to precipitate. This leads to a decrease in the number of aging precipitated phases, an increase in their size, and a reduction in their dispersion degree, resulting in a reduction in the alloy’s hardness. In addition, since these quenching precipitation phases are relatively stable, they will continue to absorb the surrounding solute atoms and grow during the following aging process, which will form a PFZ around them, as shown in Figure 5f. Compared with the matrix, PFZ is softer and lower in strength, making it more prone to deformation and dislocation accumulation, resulting in stress concentration, which is harmful to the mechanical properties of the alloy.

The effect of alloying elements on quench sensitivity is complex. An increase in Mg content within a certain range leads to substantial precipitation of quenching precipitation phases, leading to a rapid decrease in hardness and strength, thereby increasing quenching sensitivity. Similarly, an increase in Cu content raises the solid solution supersaturation and the system free energy, promoting the precipitation of more quenching phases during quenching and thus increasing quenching sensitivity. Bryant et al. [29] concluded that among the elements Zn, Mg, and Cu, Cu has the greatest effect on quenching sensitivity, followed by Mg and Zn. Chen Hui [30] found that Cu has the most significant impact on quenching sensitivity, followed by Mg, while Zn has the smallest effect, and moderately reducing Cu content and raising the Zn/Mg ratio can reduce the quenching sensitivity of the alloys in this series. The dispersed phase formed by Cr exhibits a high lattice mismatch with the Al matrix. Due to its high interfacial energy, it becomes the nucleation site for the quenching precipitation phase during slow quenching, causing high quenching sensitivity. In contrast, the Al_3_Zr phase formed by Zr has excellent coherence with the Al matrix, reducing the precipitation of quenching precipitation phases and thus effectively reducing the quenching sensitivity of the material [31]. As the content of alloying elements increases, after solid solution treatment, the concentration of solute atoms in the solid solution increases, and the degree of supersaturation increases. This makes the solid solution more prone to decomposition during quenching, leading to the rapid formation of quenching precipitation phases. In addition, the type and proportion of alloying elements can also significantly affect the precipitation behavior during quenching. These factors will change the free energy of the alloy system, precipitation driving force and quenching precipitation behavior and thus have an impact on the quenching sensitivity. Therefore, rational design of alloy composition can reduce quenching sensitivity. Based on the above theories and the accumulation of previous research by the present authors, a new high-strength 7XXX series aluminum alloy was developed.

Compared to the 7075 aluminum alloy (see Table 4 for composition [17]), the new alloy reduces the concentration of the main alloying elements Mg and Cu, decreases the content of the trace element Cr, and adds a certain amount of Zr element. In this study, the TTP curve of the new high-strength aluminum alloy is established and compared with those of 7075 and 7175 aluminum alloys for analysis. Based on Figure 9 and Table 3, it can be concluded that the new high-strength aluminum alloy exhibits the lowest quenching sensitivity among the three alloys. Additionally, the mechanical properties of the three alloys are relatively similar [17,20]. Therefore, this new alloy is suitable for the production of thick section plates and automotive hot stamping aluminum sheets.

According to the Staley quench factor analysis (QFA) theory [32], the phase transition kinetics for the continuous cooling process of aluminum alloys can be expressed as follows:

(2) $$\xi =1-exp(k_{1}\tau ),$$

In the formula, ξ is the volume fraction of the untransformed phase, k_1_ is the precipitation kinetic constant, and τ is the quench factor, which can be obtained by the following formula:

(3) $$\tau =\int_{t_{0}}^{t_{f}}\frac{1}{C_{t}(T)}dt,$$

where t is time, t_0_ is the quenching process start time, t_f_ is the quenching process termination time, and C_t_(T) is the critical transition time, which can be obtained from the TTP curves.

By combining the quenching cooling curve with the 99.5% TTP curve of the aged alloy, a prediction model for alloy performance changes is established using the QFA. The alloy hardness can be predicted by the following equation [33,34]:

(4) $$\sigma =\sigma_{max}exp(k_{1}\tau ),$$

where σ is the calculated hardness value, and σ_max_ is the maximum hardness that can be obtained for the alloy.

**Figure 10:**
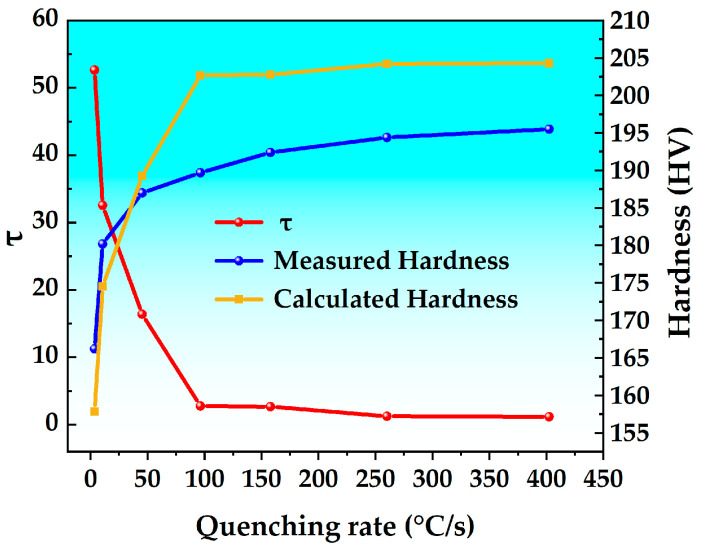
Effect of quenching rate on quenching factor and alloy hardness.

Using the experimentally measured cooling curve of the alloy (Figure 1) and the TTP curve for 99.5% transformation in the T6 state of the alloy (Figure 8), the quench factor τ is calculated in combination with Equation (3). The parameters are defined as follows: k_1_ = ln(0.995), the calculation step Δt = 0.1 s, and the calculation temperature interval is 110–500 °C. The hardness of the alloy is calculated using Equation (4) to predict the effect of the quenching rate on the hardness of the new high-strength aluminum alloy. The results are shown in Figure 10. Based on the above steps, the calculated theoretical hardness values are compared with the actual test results. The maximum difference between the theoretical and measured values is 5%, indicating that the proposed calculation method demonstrates high predictive accuracy.

As can be seen from Figure 10, as the quenching rate decreases, the quench factor τ gradually increases, and the calculated hardness value gradually decreases. When the quenching rate is 402.5 °C/s, the alloy hardness attains its maximum value, and the calculated hardness value is 204.3 HV. Further increasing the quenching rate exhibits negligible effects on hardness improvement. This phenomenon arises because solute atoms are sufficiently rapidly trapped within the supersaturated solid solution during quenching, which inhibits nucleation and precipitation. Consequently, solute atoms are retained to the greatest extent, thereby allowing the ageing strengthening effect to be fully exploited. When the quenching rate is in the range of 402.5–96.4 °C/s, the hardness of the aged alloy gradually decreases with reducing quenching rate. This is because as the quenching rate decreases, the quenched precipitation phase within the grain begins to precipitate slowly. These precipitates reduce the solute concentration in the matrix, thereby limiting the formation of strengthening phases during subsequent ageing. At a quenching rate of 96.4 °C/s, the calculated hardness of the alloy is 198.7 HV, representing a 2.7% reduction compared to the maximum hardness value. When the quenching rate Is in the range of 96.4–3.6 °C/s, the hardness exhibits a rapid decline, with the rate of decrease becoming significantly more pronounced in this range. At this time, a large number of relatively coarse quenching precipitates form within the grains, further reducing the precipitation of strengthening phases. At a quenching rate of 3.6 °C/s, the calculated hardness value is 157.8 HV, which is a 22.8% decrease from the maximum calculated hardness value.

The calculation of the quench factor τ and its correlation with alloy properties are critical for optimizing the quenching process. By comprehensively analyzing the alloy cooling curve, TTP curve, and τ, the alloy performance can be effectively predicted and optimized. For example, by reasonably controlling the quenching rate, the hardness of the alloy can be improved. Controlling a higher quenching rate in the quenching sensitive temperature range and appropriately reducing it in other ranges can effectively minimize residual stress, thereby obtaining the desired hardness value.

## 5. Conclusions

(1) When the cooling rate decreases from 402.5 °C/s to 3.6 °C/s, the hardness reduction rate is 15%. With decreasing cooling rate, the η phase precipitates at grain boundaries and within grains (on dispersed particles), consuming a significant amount of solute atoms. This results in a decrease in the quantity and an increase in the size of aging precipitates. Additionally, distinct PFZs form around grain boundaries and η phase particles, ultimately leading to the reduction of hardness.
(2) The nose temperature of the TTP curve for this novel alloy is 325 °C, with a critical transformation time of 0.4 s. The temperature range corresponding to a 99.5% peak hardness reduction on the TTP curve after isothermal holding for 10 s is 219 °C to 427 °C. Comparative analysis reveals that the new high-strength aluminum alloy exhibits lower quench sensitivity than both 7075 and 7175 alloys. Consequently, this novel alloy achieves effective quench sensitivity reduction through optimized alloying element composition design.
(3) Quench factor analysis is employed to predict the alloy hardness. The predicted values showed close agreement with experimental measurements, with a maximum deviation of approximately 5%. The smaller the quenching factor τ, the greater the hardness of the alloy after aging. By combining the relevant data of the TTP curve to optimize and control the quenching process of the alloy, the performance of the alloy can be improved. By integrating TTP curve data to optimize and control quenching rates, alloy performance is enhanced. Through developing novel high-strength aluminum alloys and optimizing their quenching processes, this research provides a theoretical basis for selecting hot forming–quenching integrated process parameters in automotive high-strength Al alloys.

## Figures and Tables

**Table 1:** The chemical composition of the new high-strength aluminum alloy (wt.%).

Element	Zn	Mg	Cu	Cr	Zr	Ti	Al
Content	5.80	2.40	1.40	0.08	0.08	0.05	Bal.

**Table 2:** k_2_–k_5_ coefficients for TTP diagram of three 7XXX series aluminum alloys.

Alloy	k_2_ (s)	k_3_ (J·mol^−1^)	k_4_ (K)	k_5_ (J·mol^−1^)	Ref.
This study alloy	8.956 × 10^−9^	10,220.6	701.9	26,052.9	
7075	1.916 × 10^−10^	1183	815.8	114,500	[17]
7175	7.6 × 10^−10^	412	750	112,200	[20]

**Table 3:** Coefficients for TTT diagram of three 7XXX series aluminum alloys.

Alloy	Critical Temperature Range (°C)	Nose Temperature (°C)	Transformation Time at Nose Temperature (s)
This study alloy	219–427 (208)	325	0.40
7075	213–428 (215)	350	0.23
7175	209–406 (197)	340	0.16

**Table 4:** The chemical composition of the 7075 aluminum alloy [17] (wt.%).

Element	Zn	Mg	Cu	Cr	Fe	Si	Al
Content	5.77	2.65	1.74	0.27	0.15	0.09	Bal.

## Data Availability

The original contributions presented in this study are included in the article. Further inquiries can be directed to the corresponding author.
